# Clinical, biochemical, and molecular profiles of three Sri Lankan neonates with pyruvate carboxylase deficiency

**DOI:** 10.1515/almed-2023-0102

**Published:** 2024-01-08

**Authors:** Eresha Jasinge, Mihika Fernando, Neluwa-Liyanage Ruwan Indika, Pyara Dilani Ratnayake, Nalin Gamaathige, Ratnanathan Ratnaranjith, Sabine Schroeder, Patricia Jones, Skrahina Volha, Subhashinie Jayasena, Anusha Varuni Gunaratna, Asitha Niroshana Bandara Ekanayake, Arndt Rolfs

**Affiliations:** Department of Chemical Pathology, Lady Ridgeway Hospital for Children, Colombo 8, Sri Lanka; Department of Biochemistry, Faculty of Medical Sciences, University of Sri Jayewardenepura, Nugegoda, Sri Lanka; Department of Neurology, Lady Ridgeway Hospital for Children, Colombo 8, Sri Lanka; Neonatal Intensive Care Unit, De Soysa Hospital for Women, Colombo 8, Sri Lanka; District General Hospital, Vavuniya, Sri Lanka; CENTOGENE AG, Rostock, Germany; Children’s Medical Center, University of Texas Southwestern Medical Center, Dallas, TX, USA; University of Rostock, Rostock, Germany

**Keywords:** genotype, citrulline, neonate, phenotype, pyruvate carboxylase deficiency

## Abstract

**Objectives:**

Pyruvate carboxylase, a mitochondrial enzyme, catalyses the conversion of glycolytic end-product pyruvate to tricarboxylic acid cycle intermediate, oxaloacetate. Rare pyruvate carboxylase deficiency manifests in three clinical and biochemical phenotypes: neonatal onset type A, infantile onset type B and a benign C type. The objective of this case series is to expand the knowledge of overlapping clinical and biochemical phenotypes of pyruvate carboxylase deficiency.

**Case presentation:**

We report three Sri Lankan neonates including two siblings, of two unrelated families with pyruvate carboxylase deficiency. All three developed respiratory distress within the first few hours of birth. Two siblings displayed typical biochemical findings reported in type B. The other proband with normal citrulline, lysine, moderate lactate, paraventricular cystic lesions, bony deformities, and a novel missense, homozygous variant c.2746G>C [p.(Asp916His)] in the *PC* gene, biochemically favoured type A.

**Conclusions:**

Our findings indicate the necessity of prompt laboratory investigations in a tachypneic neonate with coexisting metabolic acidosis, as early recognition is essential for patient management and family counselling. Further case studies are required to identify overlapping symptoms and biochemical findings in different types of pyruvate carboxylase deficiency phenotypes.

## Introduction

Pyruvate carboxylase (PC; EC: 6.4.1.1) is a mitochondrial enzyme that catalyzes the ATP-dependent carboxylation of pyruvate to oxaloacetate [1]. PC, a biotin-containing homotetramer arranged in a tetrahedron-like structure [2], which is allosterically activated by acetyl-CoA, is encoded by the gene located on the long arm of chromosome 11 [3, 4]. PC is mainly responsible for anaplerotic activity by replenishing the intermediates of the tricarboxylic acid cycle via oxaloacetate in addition to being a regulator in other pathways involving gluconeogenesis, lipogenesis, amino acid and neurotransmitter production [5].

Pyruvate carboxylase deficiency (PCD; MIM# 266150), an autosomal recessive disorder with an estimated incidence of one in 250,000 births [6], manifests as three clinical phenotypes: type A (American type or infantile form) usually presents several months after birth with developmental delay, hypotonia, failure to thrive, mild to moderate lactic acidosis and has more prolonged survival. Type B (French type or neonatal form) has a severe outcome and manifests primarily during the first 72 h of life with tachypnea and severe truncal hypotonia [7, 8]. Type C (intermittent or benign form) presents during the first year of life with episodes of metabolic acidosis during physiological stress with normal or mild intellectual delay [9, 10]. Biochemical findings, although not pathognomonic may help differentiate these three phenotypes: high plasma lactate often >10 mmol/L, citrulline and lysine levels are often seen in patients with type B phenotype while in type A and type C citrulline levels are normal [11]. The diagnosis of PCD relies on the enzyme levels performed on cultured skin fibroblasts, lymphoblasts and/or detection of *PC* gene variants [11, 12]. The residual enzyme activity cannot be used to differentiate between the three main phenotypes but it influences the severity of clinical presentation [6, 7, 10]. The phenotype and genotype correlation has shown a high prevalence of missense mutations in type A and truncating mutations in type B [11].

The main aim of this study is to describe the biochemical patterns of three Sri Lankan patients with neonatal-onset PCD and to highlight the genotype, hitherto not reported, in one neonate with normal citrulline levels.

## Case presentation

We recruited three neonates of two unrelated Sri Lankan families (A and B) referred to the department of chemical pathology, Lady Ridgeway Hospital for Children (LRH), for the analysis of plasma amino acids and urine organic acids, encountered over seven years (2014–2020). Informed written consent was obtained from the parents.

Routine biochemical investigations in serum and lactate in plasma were performed on automated biochemistry analyzers. Samples for plasma amino acids were collected into EDTA containers, immediately centrifuged, deproteinised and analysed by high-pressure liquid ion-exchange chromatography technique (HPLC) using PerkinElmer amino acid analyser combined with Pickering post-column ninhydrin derivatisation system at the Department of Chemical Pathology of LRH. Reagents, standards and quality control samples were purchased from Sigma Aldrich.

Organic acids were extracted using previously optimized methods [13] and were analyzed qualitatively using gas chromatography-mass spectrometry (GC-MS) system by Agilent. All organic solvents and other reagents were of analytical grade.

The urine sample of patient 2 from family A, was stored and transported as per guidelines and assayed for amino acids by Biochrome System and organic acids by GC-MS (Agilent) in Dallas Children Hospital laboratory, Texas, USA, before the establishment of those tests in the department of chemical pathology LRH in 2017. Blood and urine samples of the other two patients were analyzed for plasma amino acids and urine organic acids in the department of chemical pathology LRH.

Dried blood samples of patients 2 and 3 were assayed by liquid chromatography-tandem mass spectrometry (LC-MS/MS) in the NeoGen Labs Pvt. Ltd, India. *PC* gene was analysed by Sanger sequencing of the polymerase chain reaction products obtained from peripheral DNA at CENTOGENE AG, Germany. Reference sequence of the *PC* gene is NM_000920.3.

### Family A

**Figure 1:**
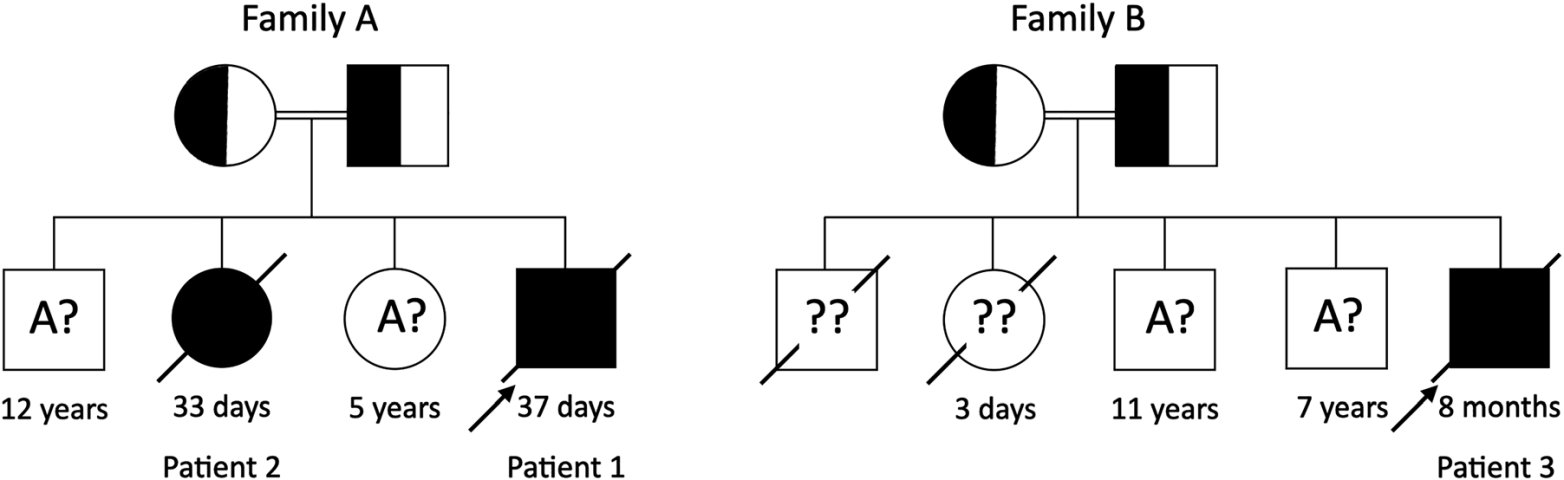
Pedigree charts of Family A and Family B. The families include unaffected siblings as well as diseased siblings whose exact genotypes are unknown (A? and ?? respectively). The diseased siblings are likely to be homozygous (aa) for the pathogenic variants. A: dominant allele, a: recessive allele, ?: unknown allele.

Patients 1 and 2 are products of healthy consanguineous Sri Lankan parents with no significant family history. Patient 1 was genetically confirmed to have PCD. Parents have two surviving healthy children aged 12 and 5 years. Two neonates succumbed to their illness at the age of 33 and 37 days (Figure 1).

#### Patient 1

A one-day-old baby boy, the fourth child of the family was born to consanguineous parents at term following an uncomplicated pregnancy with a birth weight of 2.190 kg (<−3SD) (Table 1). There was a history of a sibling death on day 33 of life with a questionable biochemical diagnosis of argininosuccinate synthase deficiency.

**Table 1:** Demographic and clinical features.

	Patient 1 [Family A]	Patient 2 [Family A]	Patient 3 [Family B]
Age at onset	3 h after birth	At birth	Within an hour of birth
Sex	Male	Female	Male
Consanguinity	Yes	Yes	Yes
Affected siblings	Yes (patient 2)	Yes (patient 1)	Yes, two neonatal deaths
Pregnancy complications	None	None	Gestational diabetes
Maturity at birth	Term	Term	34 weeks
Mode of delivery	Vaginal	Vaginal	Caesarian section
Birth weight, kg	2.19 (<−3SD)	2.05 (−2SD to −3SD)	2.05 (<−3SD)
Age at death	Day 37	Day 33	8 months

His birth was uneventful; however, he developed tachypnoea 3 h after birth requiring admission to a Special-Care Baby Unit (SCBU). Upon examination, he had blond hair, acidotic breathing, mild hepatomegaly and generalized hypotonia with preserved reflexes. Blood gas level on admission showed metabolic acidosis (pH 7.12, HCO_3−_ 4.2 mmol/L). His capillary glucose was 38 mg/dL by a point-of-care glucometer, plasma lactate was 18.75 mmol/L (0.5–2.2), and urine ketone bodies were positive. He had recurrent convulsions while in the hospital, which were successfully managed with antiepileptics including levetiracetam and phenobarbitone. Plasma amino acid analysis (µmol/L) performed on day two of life showed high citrulline, alanine, lysine and tyrosine (Table 2). The urine organic acid profile elicited very high lactate, 3-hydroxybutyrate (beta-hydroxybutyrate), acetoacetate, 2-hydroxyburtyrate, 4-hydroxyphenyllactate, mild amounts of 4-hydroxyphenylpyruvate, fumaric and 2-hydroxyglutarate.

**Table 2:** Amino acid results in µmol/L (whole blood, plasma and urine).

Amino acid	Reference ranges			Patient 1 [Family A]	Patient 2 [Family A]		Patient 3 [Family B]	
	Plasma	DBS	Urine	Plasma	Urine	DBS	Plasma	DBS
Alanine	131–710	<960	767–6,090	**1,086**	323	241.19	448	456.2
Citrulline	3–55	3–75	22–181	**243**	**1,165**	**113.6**	17	23.7
Lysine	92–325	NP^a^	189–850	**861**	40	NP^a^	175	NP^a^
Tyrosine	22–147	<300	20–1,650	**384**	62	214.94	85	81.1
Aspartate	20–129	9–108.1	0–240	**5**	2	14.25	18	44.4
Glutamate	62–620	19–265.83	55–590	**24**	**20**	39.08	70	48.76
Glutamine	200–1,200	58–450	393–1,562	**135**	**20**	54.99	253	246

The pertinent abnormal values are indicated in boldface. ^a^NP, not performed.

Though citrullinemia type 2 was considered as a differential diagnosis given the moderate citrulline levels, high alanine and altered liver function tests (Table 2), PCD was regarded as the principal differential diagnosis due to severe lactic acidosis along with high citrulline, alanine and lysine in plasma. Genetic analysis of the *PC* gene revealed a nonsense homozygous, most likely a pathogenic variant c.2514G>A [p. (Trp838*)] confirming the diagnosis of PCD. He was managed conservatively with bicarbonate replacement, vitamin B12, biotin, carnitine, co-enzyme-Q, thiamine, pyridoxine, aspartate, and sodium citrate/citric acid (bicitra).

After a month of treatment with normal routine biochemical markers, he was discharged. The baby continued to be breast fed. Unfortunately, the baby died post one week of discharge with a possible history of milk aspiration.

Upon the genetic confirmation of PCD, the history of the sibling (patient 2) was reviewed.

#### Patient 2

The female proband was born at term, via spontaneous vaginal delivery, after an uneventful antenatal period as the second child of family A. Her birth weight was 2.05 kg (−2SD to −3SD). Poor activity, refusal of feed and respiratory distress were noted since birth. On day-10 she became severely acidotic (pH 7.21, HCO_3−_ 7.3 mmol/L) (Table 3) with pronounced ketonuria. A dried blood sample analysed by tandem mass spectrometry revealed a high citrulline level (113.6 μmol/L). Urine amino acid analysis showed increase citrulline (1,165 μmol/L) and absent argininosuccinate (Table 2). Urine organic acid profile demonstrated marked ketosis (3-hydroxybutyrate>acetoacetate) and massive excretion of lactate, 3-phenyllactate, 2-hydroxyisobutyrate, 4-hydroxyphenylacetate and 4-hydroxyphenylpyruvate.

**Table 3:** Routine biochemical analysis at the onset of symptoms.

Analyte	Unit	Reference range	Patient 1 [Family A]	Patient 2 [Family A]	Patient 3 [Family B]
Plasma lactate	mmol/L	0.5–2.3	**18.75**	NP^a^	**4–9.6**
Venous blood gas					
pH		7.33–7.44	**7.12**	**7.32**	**7.24**
HCO_3−_	mmol/L	23–28	**4.2**	**8.6**	**6.1**
Serum biochemical markers					
Total bilirubin	µmol/L	3–20	**61**	NP^a^	**134**
Direct bilirubin	µmol/L	0–3	**27**	NP^a^	NP^a^
Alkaline phosphatase	IU/L	60–425	**443**	NP^a^	153
Aspartate transaminase	IU/L	0–40	**64**	**377**	**113**
Alanine transaminase	IU/L	9–48	39	**301**	30
Gammaglutamyl transferase	IU/L	2–30	**211**	NP^a^	NP^a^
C reactive protein	mg/L	<5.0	34	23	NP^a^
Creatine kinase	IU/L	28–300	**1,228**	NP^a^	181
Urea	mmol/L	1.0–2.5	**7.9**	**4**	**3.9**
Creatinine	µmol/L	35–40	**75**	16	**45**
Total cholesterol	mmol/L	1.09–2.07	**2.58**	NP^a^	1.69
Triglycerides	mmol/L	0.97–3.13	**4.5**	NP^a^	0.95

Increased lactate levels (hence metabolic acidosis) and lipid levels are secondary to accumulation of pyruvate and acetyl-CoA respectively. Increased bilirubin and liver enzymes indicate hepatocellular dysfunction. The pertinent abnormal values are indicated in boldface. ^a^NP, not performed.

A diagnosis of argininosuccinate synthase deficiency was based mainly on the results of blood and urine amino acid profiles. Genetic confirmation was not feasible at that point in time. She was managed in the SCBU with intravenous fluids and bicarbonate. Her weight on day-21 was 1.81 kg (<−3SD). By day-33 of life the baby succumbed to her illness. Retrospective analysis of the clinical and biochemical profiles in a neonate presenting with respiratory compromise, severe metabolic acidosis, along with high citrulline and lactic aciduria, supported by the genetic results of the sibling (patient 1), allowed us to consider PCD as the diagnosis in this patient as well.

### Family B

Patient 3 is the product of a consanguineous Sri Lankan family. The first two children, a boy and a girl, died on the third day of life following respiratory distress. The family has two healthy survivors of eleven and seven-years old (Figure 1).

#### Patient 3

The male patient was born as the fifth child by emergency Caesarean section at 33+3 weeks of gestation owing to past Caesarean section. Except for gestational diabetes in the 39-year-old mother of the proband, pregnancy was uncomplicated.

His 1, 5 and 10 min Apgar scores were 9–10–10 respectively with birth weight 2.05 kg (<−3SD), length 48 cm (−1SD) and head circumference 33 cm (−1SD to median). As he developed respiratory distress and tachypnea within 1 h of delivery, he was admitted to a SCBU. His blood gas results at 3 h of life showed metabolic acidosis (pH 7.27, pCO_2_ 42 mmHg, HCO_3−_ 18.3 mmol/L, lactate 4 mmol/L). The patient was treated with intravenous bicarbonate and 2 L of nasal canula oxygen. Second venous blood gas performed at 7 h of life indicated an improvement in parameters except high level of lactate (pH 7.41, pCO_2_ 26.8, pO_2_ 66, HCO_3−_ 16.8, lactate 4.6). By day-4 of life, there was a clinical improvement and the child was transferred and observed at the postnatal ward. Additionally, neonatal examination revealed right lower limb deformity (fibula hemimelia with second toe syndesmosis with underdeveloped foot) and overriding cranial sutures.

He was readmitted to SCBU at day-7 of life due to less activity, poor sucking, respiratory distress and poor weight gain (12 % weight loss). A hypoglycaemic episode on admission (capillary blood glucose level was 36 mg %) was treated with an intravenous bolus of 10 % dextrose. As metabolic acidosis continued (pH 7.24, pCO_2_ 6, lactate 4.6, HCO_3−_ 6.1) a bicarbonate correction was performed. Subsequent blood gases revealed an improvement in pH and bicarbonate levels but the rise in lactate continued (9.6 mmol/L). Plasma amino acid analysis of the sample obtained on day-8 of life showed normal citrulline, alanine and lysine levels. Simultaneous screening of the DBS sample by tandem mass spectrometry revealed a normal amino acid profile (Table 2). The urine organic acid profile depicted very high lactate, mild elevation of 4-hydroxyphenyllactate, 3-hydroxybutyrate, acetoacetate, fumarate and 2-hydroxyglutarate. Nasal cannula oxygen and antibiotics were restarted. Intravenous sodium bicarbonate correction was carried out thrice. Later it was converted to oral sodium bicarbonate (2 mmol/kg/day in two divided doses). After 3 days of readmission baby clinically improved, but persistently high lactate was noted with metabolic acidosis. Right-sided mild pneumothorax observed in the chest X-ray resolved spontaneously.

On discharge at the age of 19-days, he was clinically, and biochemically stable though lactate was mildly elevated (pH 7.4, pCO_2_ 30.3, pO_2_ 71, HCO_3−_ 22.3, lactate 3.1). After the initial reduction in weight to 1.78 kg, a physiological phenomenon, he regained weight at a rate of 15 g/day to reach 1.86 kg on discharge. Both breast and cup feeding were established with added iron and multivitamin supplements.

Child was regularly followed up in paediatric and orthopaedic clinics. At the age of 2-months, he weighed 2.8 kg (−2SD to −3SD), height of 53 cm (−1SD to −2SD) and head circumference of 37 cm (+1SD to +2SD). Ultrasound scan of the brain performed as a routine scan for a premature baby elucidated porencephalic cysts in the frontal lobe adjacent to the anterior horn, right side cyst−12.5×15.1 mm and left side cyst−12.5×17.2 mm while posterior fossa and bilateral ventricles were normal.

By 6-months global developmental delay was evident while anthropometry revealed slight gain in weight [6.8 kg (−1SD to −2SD)] without an improvement in height [65 cm (−2SD to −3SD)] and remarkable microcephaly of 41 cm (−2SD to −3SD).

A missense, homozygous variant c.2746G>C [p.(Asp916His)] was found in the *PC* gene giving a diagnosis of PCD. The variant is classified as uncertain significance according to the recommendations of CENTOGENE and American College of Medical Genetics and Genomics (ACMG).

At the age of 8 months, he developed a lower respiratory tract infection which pushed the equilibrium towards severe refractory metabolic acidosis. He passed away despite intensive care in a tertiary care center.

## Discussion

We describe the clinical, biochemical and molecular findings of three patients with PCD. Demographic and clinical presentation of three patients seem to be more homogenous, being born to consanguineous parents with affected siblings, low birth weights and early onset of respiratory distress.

Prenatal growth retardation manifested as low birth weights in all three neonates is a probable finding in neonatal energy metabolism defects, though normal birth weights have been described in previous reports [14, 15]. Bone deformities observed in patient 3, a finding that has not been reported, might be an occasional finding yet it deserves attention.

Biochemical abnormalities such as metabolic acidosis, high plasma lactate [14, 15] and elevated transaminases [14] described in the literature were disclosed in our patients. Mild increase in direct bilirubin, alkaline phosphatase and high gamma glutamyl transferase were additional biochemical findings noted in patient 1. The mildly elevated cholesterol in the same patient could be due to the diversion of excess ketone bodies to produce acetyl CoA and acetoacetyl CoA as described in PCD types A and B [16].

Hypoglycaemia observed in patients 1 and 3, an expected finding due to direct involvement of PC in gluconeogenesis [11], has been described in the literature [14]. The abnormalities in the amino acid profile of the patient 1 were clearly related to PCD. For instance, high citrulline is due to deficient oxaloacetate and consequently aspartate, an amino acid required to synthesize argininosuccinate from citrulline. This is a frequent occurrence in patients with type B [11]. This was evident in the analysis of both siblings’ DBS, plasma or urine. Lysine elevation and low glutamate (hence low glutamine) levels in patient 1 can be explained by deficiency of alpha-ketoglutarate (2-oxoglutarate) required for lysine breakdown and glutamate production. Alanine levels can increase due to transamination of accumulated pyruvate. The suspicion of PCD was strong when considering together the results of urinary organic acids (high lactate, acetoacetate, 3-hydroxybutyrate) and the hypoglycemia. But, argininosuccinate synthase deficiency (ASSD) is characterized by elevated ammonia, glutamine, and citrulline [17], therefore low blood glutamine levels in patient 1 makes ASSD diagnosis unlikely. Hyperammonemia is also a metabolic phenotype of PCD type B [18]. Increased tyrosine level observed in patient 1 is a less commonly reported finding in PCD type B [19].

**Figure 2:**
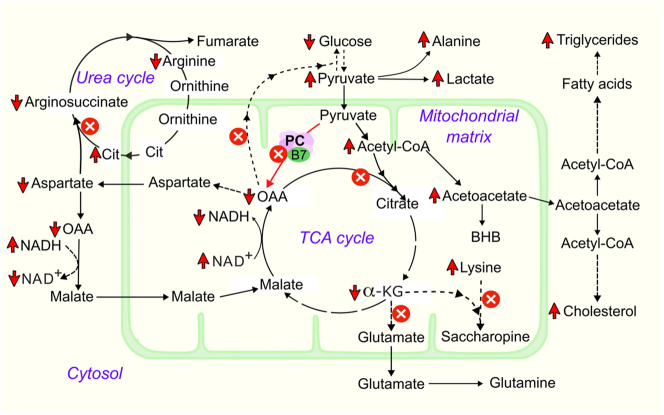
Biochemical changes associated with pyruvate carboxylase deficiency; the intermediates of the tricarboxylic acid (TCA) cycle are decreased. Gluconeogenesis is impaired due to reduced oxalate availability. The urea cycle is impaired due to aspartate deficiency resulting in hypercitrullinemia. Lysine degradation may be impaired due to alpha-ketoglutarate deficiency. Increased acetyl-CoA is directed towards ketogenesis, fatty acid synthesis, and cholesterol synthesis. Cytoplasmic NAD^+^/NADH ratio is decreased while mitochondrial NAD^+^/NADH ratio is increased. α-KG, alpha-ketoglutarate; B7, biotin; BHB, beta-hydroxybutyrate; Cit, Citrulline; HMG-CoA, β-hydroxy β-methylglutaryl-CoA; NAD^+^, oxidized nicotinamide adenine dinucleotide; NADH, reduced nicotinamide adenine dinucleotide; OAA, oxaloacetate; PC, pyruvate carboxylase.

Biochemical and clinical presentation enabled us to consider patient 1 and 2 to have PCD type B. Patient 2 was initially diagnosed to have ASSD based solely on high whole blood and urinary citrulline in the absence of argininosuccinate, despite whole blood citrulline levels not being in the expected range as observed in ASSD. The diagnosis of PCD was retrospectively made when it was diagnosed in the sibling (patient 1). This case highlights the challenges that occur in the diagnosis when required investigations are not available, mainly plasma lactate another important test that is not widely available to date in most of the laboratories in Sri Lanka. High lactate is a characteristic finding in patients with PCD [6, 14]. The biochemical changes associated with PCD are illustrated in Figure 2.

Citrulline level in patient 3 analysed by two different analytical principles namely tandem mass spectrometry in whole blood and high-performance liquid chromatography in plasma, gave normal results. Though his neonatal onset favours PCD type B, plasma lactate (<10 mmol/L) and normal citrulline levels do suggest PCD type A. Literature describes a PCD type A in a neonate with normal citrulline and lysine levels [20].

High lactate, 4-hydroxyphenylacetate and 4-hydroxyphenyllactate were a constant finding in the urine organic acid profiles of all three patients. Elevated urinary 4-hydroxyphenylacetate and 4-hydroxyphenyllactate levels have been revealed by a recent case report [19]. In addition, ketotic compounds were in moderate amounts with low levels of tricarboxylic acid compounds. Most published data indicate increased urinary levels of 2-hydroxybutyrate, urine 3-hydroxybutyrate, and acetoacetate, and reduced urinary levels of tricarboxylic acid compounds such as alpha-ketoglutarate (2-oxoglutarate) fumarate, oxaloacetate and malate [11, 14].

Patients with PCD type B also exhibit increased pyruvate, lactate/pyruvate ratio and acetate/3-hydroxybutyrate ratio in blood [18]. However, plasma pyruvate, acetoacetate or 3-hydroxybutyrate assays are not available in Sri Lanka, hence the ratios couldn’t be calculated in our patients.

All three patients described in the resent case series exhibited several abnormalities in the general biochemical investigations such as elevated serum bilirubin, aspartate transaminase, alkaline phosphatase and creatine kinase. Interestingly similar findings were reported by case report of a neonate with PCD type B [19].

Symmetrical paraventricular cavities around the frontal and temporal horns of the lateral ventricles, a finding observed in patient 3, has been described in the literature [21, 22]. Deficient lipogenesis due to lack of cytosolic oxaloacetate may be the possible explanation for this finding as well as for widespread demyelination of cerebral and cerebellar white matter.

Both siblings though they seem to harbour the same mutation in homozygosity, their biochemical findings show heterogeneity. Complex missense mutations, splice donor site mutations, deletions in homozygosity, along with compound heterozygosity and mosaicism have been described as phenotypes of PCD type B in literature. In contrast, patients with PCD type A frequently harbor two missense mutations in homozygosity or compound heterozygosity [11, 15, 18, 23]. The mechanisms underlying such biochemical and genetic variability are unclear. They could be multifactorial: the amount of PC protein, remaining enzyme activity in tissues, genetic variant, and Influence on environmental factors [23].

The PC variant c.2514G>A p.(Trp838*) of patient 1, and presumably of patient 2, which creates a stop codon explains the severity of the phenotype in contrast to the missense novel variant c.2746G>C p.(Asp916His) causing an amino acid change from aspartate to histidine at position 916 of patient 3. Clinical and biochemical findings of both neonates from family A are consistent with PCD type B while patient 3 though onset is neonatal, biochemical and molecular findings do favour type A.

Our study confirms the already reported finding of clinical presentation and outcome though some of the findings seem to be novel. The results of our study will bring awareness among clinicians about the existence of this rare disorder and the need to perform relevant investigations.

## Lessons learned

– Plasma lactate, plasma amino acid profile and organic acid profile are helpful investigations to arrive at a tentative diagnosis of PCD in a developing country.
– Elevation of serum aspartate transaminase, alkaline phosphatase and creatine kinase could be biochemical abnormalities associated with PCD type B.
– Urinary lactate, 4-hydroxyphenylpyruvate, and 4-hydroxyphenyllactate levels were consistently elevated in all three cases.
– When high plasma lactate and ketonuria are observed in a neonate with respiratory distress, PCD should be suspected.
